# Starch biosynthetic genes and enzymes are expressed and active in the absence of starch accumulation in sugar beet tap-root

**DOI:** 10.1186/1471-2229-14-104

**Published:** 2014-04-23

**Authors:** Helle Turesson, Mariette Andersson, Salla Marttila, Ingela Thulin, Per Hofvander

**Affiliations:** 1 Department of Plant Breeding, Swedish University of Agricultural Sciences, P.O. Box 101, SE-23053 Alnarp, Sweden; 2 Department of Plant Protection Biology, Swedish University of Agricultural Sciences, P.O. Box 102, SE-23053 Alnarp, Sweden; 3 SOLAM AB, Degebergavägen 60-20, SE-291 91 Kristianstad, Sweden

**Keywords:** *Beta vulgaris*, *Pastinaca sativa*, Storage accumulation, Carbon allocation, Starch, Sucrose

## Abstract

**Background:**

Starch is the predominant storage compound in underground plant tissues like roots and tubers. An exception is sugar beet tap-root (*Beta vulgaris ssp altissima*) which exclusively stores sucrose. The underlying mechanism behind this divergent storage accumulation in sugar beet is currently not fully known. From the general presence of starch in roots and tubers it could be speculated that the lack in sugar beet tap-roots would originate from deficiency in pathways leading to starch. Therefore with emphasis on starch accumulation, we studied tap-roots of sugar beet using parsnip (*Pastinaca sativa*) as a comparator.

**Results:**

Metabolic and structural analyses of sugar beet tap-root confirmed sucrose as the exclusive storage component. No starch granules could be detected in tap-roots of sugar beet or the wild ancestor sea beet (*Beta vulgaris ssp. maritima*). Analyses of parsnip showed that the main storage component was starch but tap-root tissue was also found to contain significant levels of sugars. Surprisingly, activities of four main starch biosynthetic enzymes, phosphoglucomutase, ADP-glucose pyrophosphorylase, starch synthase and starch branching enzyme, were similar in sugar beet and parsnip tap-roots. Transcriptional analysis confirmed expression of corresponding genes. Additionally, expression of genes involved in starch accumulation such as for plastidial hexose transportation and starch tuning functions could be determined in tap-roots of both plant species.

**Conclusion:**

Considering underground storage organs, sugar beet tap-root upholds a unique property in exclusively storing sucrose. Lack of starch also in the ancestor sea beet indicates an evolved trait of biological importance.

Our findings in this study show that gene expression and enzymatic activity of main starch biosynthetic functions are present in sugar beet tap-root during storage accumulation. In view of this, the complete lack of starch in sugar beet tap-roots is enigmatic.

## Background

Plants produce and store energy reserves for various purposes. A major use of these energy reserves is to facilitate growth and propagation of the next generation and they are laid down in sink tissues, e.g. seeds and tubers. The plant storage reserves, starch, oil and sugars, are supplying mankind with the majority of calories but have also important industrial applications. The type of storage compound and in which tissue of the plant the storage product is located varies among plant species. Generally, the biosynthesis of storage compounds, starch, oil and sugars, is known in quite detail but the knowledge of why a certain type of these products accumulates and the underlying mechanisms are largely lacking [1,2]. With increased knowledge of key points governing the accumulation of a certain storage compounds in a storage sink, plants might be tailored for increased accumulation and yield. Alternatively, plants might be engineered to accumulate additional storage compounds than naturally occurring.

In general, tap-roots have starch biosynthetic and deposition capacity and starch granules can readily be found in cells of parsnips, carrots and swedes. An exception among tap-roots is sugar beet (*Beta vulgaris ssp. altissima*) and related subspecies which produce no starch but sucrose during tap-root development. The reason why beets exclusively have sucrose as a storage compound is not known. However, one factor that might have been of importance are the saline growth conditions where the wild ancestor, sea beet (*Beta vulgaris ssp. maritima*), grows. Sea beet is growing, as the name implicates, by the sea, and can have both an annual and biennial life cycle and has a similar cell organization and storage accumulation as sugar beet [3]. Sugar beet has a biennial life cycle with an initial tap-root the first year that stores energy utilized for bolting, flowering and seed setting the second year. Sea beet tap-root was early known to be rich in sugar and was established as a source of sugar when extraction from beets was started in the beginning of the 19th century [4]. Through breeding, sugar beet has become a plant with a large tap-root containing 65-75% of sugar of the dry weight [5,6] and is today one of our major sources of sugar.

The development of underground storage tissues, such as tap-roots and tubers, display a similar cycle of temporal events regarding transport of sucrose into the cell, building of the cell components and expansion of the storage organ. Initially, apoplastic unloading of sucrose is dominating and cell wall bound acid invertase splits sucrose into hexoses which are used for growth and metabolism [7,8]. When organ developmental stage transitions to filling of energy reserves in the cells, sucrose import switches to symplastic loading. During this phase, plants activate different routes of syntheses and fill organelles with carbon compounds in the form of starch in the amyloplast or sucrose in the vacuole. Sucrose translocation and storage in sugar beet tap-root has been investigated [9,10]. In contrast to starch storage in amyloplasts, the storage of sucrose in vacuoles will, due the osmotic potential created, require a continuous energy input in order to maintain the much higher concentration of sucrose in this organelle compared to the cytosol. The membrane potential to maintain this difference in concentrations is carried out by proton pumps that utilize ATP and pyrophosphate (PPi) [11,12].

Starch biosynthesis takes place in underground tissues such as roots and tubers in a plastid dedicated to produce starch, the amyloplast [13]. Whereas sucrose is the same molecule that is transported from source tissues and thus theoretically needs no further modifications before storage in a vacuole, starch needs a number of enzymatic steps for its formation. For dicotyledons, there are four enzymatic steps that are essential in the formation of a starch polymer after the entry of glucose-6-phosphate via glucose-6-phosphate transporter (GPT) into the amyloplast [14]. A plastidic phosphoglucomutase converts incoming glucose-6-phosphate to glucose-1-phosphate, which together with ATP can be used by ADP-glucose pyrophosphorylase for the formation of ADP-glucose. ADP-glucose is the basic building block that is used by different forms of starch synthases to form the α-1,4 linkages in the polymeric chains of starch. Starch branching enzyme catalyzes the formation of α-1,6 linkages creating branches to the polymeric chains. No net starch is produced by the starch branching enzyme, but it is of importance for structuring the amylopectin [15]. Additional enzymes with starch tuning abilities, as isoamylase and starch phosphorylase, are needed for the building and organization into well-structured starch granules [14].

Production of starch in sugar beet leaves during photosynthesis as part of the diurnal cycle demonstrates that all genes central for starch biosynthesis are present in sugar beet, like in all other plants [16]. The same conclusion can be made from searching public databases of sugar beet expressed sequence tags (ESTs). A few studies on starch biosynthesis and responsible enzymes have been performed on sugar beet leaves [17-19]. However studies on gene expression or enzyme activities related to starch biosynthesis in sugar beet tap-root have to our knowledge not yet been reported.

The aim of this study was to, during a developmental cycle, investigate the nature of the storage compounds and to what extent genes and enzymes central to starch biosynthesis are manifested in tap-roots of sugar beet and parsnip (*Pastinaca sativa*). Sugar beet and parsnip root have similar behaviour and morphology but the main storage compounds of the two tap-roots differ. Sugar beet and parsnip were grown in a greenhouse and samples were taken at two different developmental time points. At these two time points samples were taken both at the end of the light- as well as at the end of the dark period of the day to monitor potential diurnal changes. Roots from the two plant species were comparatively studied with focus on carbon allocation as well as expression of genes essential for starch accumulation and activities of the main starch biosynthetic enzymes.

Storage compound analysis of sea beet was included in the study to analyse if the lack of starch is a conserved trait from this wild ancestor. Our results show transcription of essential starch biosynthetic genes and presence of active starch biosynthetic enzymes but no starch is accumulated in sugar beet tap-root. This implies that lack of starch in sugar beet tap-root and its carbon allocation is not a simple loss of gene functions in pathways leading to starch.

## Results

### Structural studies

Structural studies were performed in order to compare the different species and subspecies visually on a cellular level. This was done to confirm other measurements, such as compositional studies and with temporally differentiating samples to verify that the material was in a storage accumulation phase. Root and leaf tissue of parsnip and sugar beet were embedded in plastic and sections were studied by both general and specific histochemical staining.

**Figure 1:**
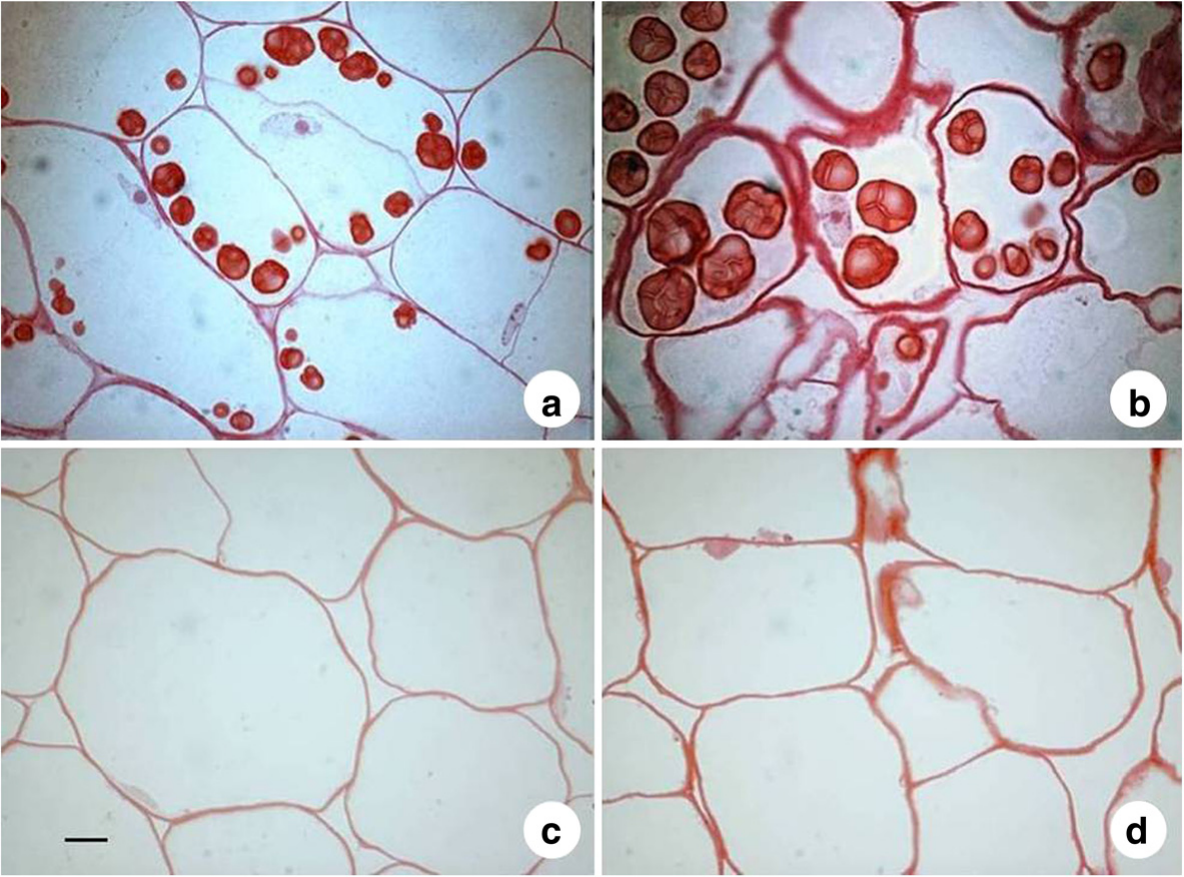
**Light micrographs of parsnip and sugar beet tap-root storage tissue.** Sections of parsnip 48 days after planting **(a)**, parsnip 61 days after planting **(b)**, sugar beet 41 days after planting **(c)** and sugar beet 54 days after planting **(d)** were stained with MAS (Triple staining methylene blue-azur A-safranin O). Scale bar 10 μm.

The studies showed that sugar beet and parsnip tap-root cells have structural similarities in the early development of the cells prior to active storage accumulation (Figure 1). Early stage tap-root cells were found to be vacuolized in both parsnip and sugar beet and the cell size was already large when compared to later stages. In parsnip tap-root small initial starch granules were displaced to the periphery of the cell by the vacuole that occupied most of the space in the cell. With further development, parsnip root cells accumulated more starch via enlargement of starch granules. No starch granules could be detected in sugar beet tap-root cells in either of the studied harvest time points. There was no apparent difference in cell size between the different samples of the sugar beet tap-root cells but that cell walls thickened during development. Especially in the late samples, β-glucans had accumulated in the cell walls (results not shown). The vacuoles of sugar beet root cells maintained their relative size during growth contrasting to parsnip where the growing starch granules occupied more and more of the cell volume (Figure 1).

Homogenized tap-root tissue of sea beet, the origin of sugar beet, was examined for starch granules. Light microscopical examination did not reveal any structures resembling starch granules (results not shown).

The leaves of parsnip and sugar beet appeared to behave as any other photosynthetic source tissue. Leaf tissue displayed diurnal changes with excess sucrose produced during photosynthesis stored as starch during the light period and subsequently degraded with sucrose resynthesized and transported to other parts of the plant during the dark period (Additional file 1).

### Temporal tap-root development and storage compound accumulation

**Figure 2:**
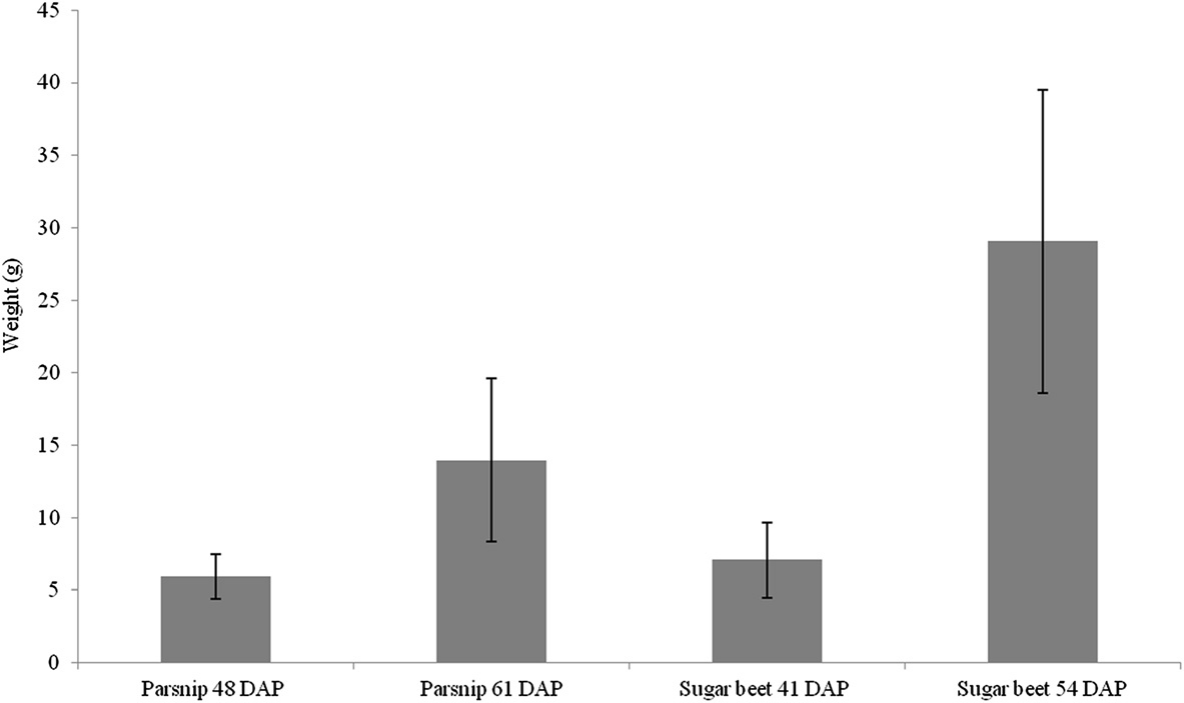
**Average fresh weight of parsnip and sugar beet tap-roots.** Parsnip tap-roots were harvested 48 and 61 days after planting (DAP) and sugar beet tap-roots harvested 41 and 54 DAP (n = 40). Vertical bars correspond to the standard deviation of the average.

Fresh and dry weights were measured at two different time points to determine that the sampled roots were in a phase with an ongoing accumulation. On tissue sections of the sampled sugar beet roots, 3–5 secondary cambium rings could be seen (Additional file 2). A mature sugar beet root consists of about 12 secondary cambium rings, where the first 8 cambium rings develop during the first 8 weeks [20]. Our results verified that the plants sampled were an appropriate material for this study. Tap-roots were sampled at the end of a light period as well as at the end of a subsequent dark period. The results of individual weight measurements were not considered since no differences could be found in fresh weight between harvests taken after the light period compared to after the subsequent dark period. As expected, fresh weights of tap-roots for both species increased over time (Figure 2). Dry weights of tap-roots in parsnip tap-root increased from an average of 14% to an average of 20% whereas for sugar beet tap-root there was a similar increase in dry weight from 15% to 19% (results not shown).

### Sugars and starch in tap-roots of sugar beet and parsnip

**Figure 3:**
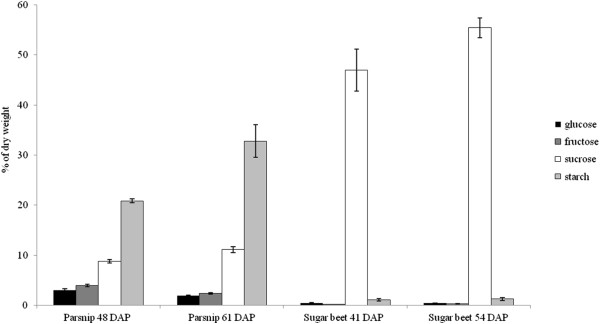
**Starch and sugars content in parsnip and sugar beet tap-roots.** Parsnip tap-roots harvested 48 and 61 days after planting (DAP) and sugar beet tap-roots harvested 41 and 54 DAP. Results are reported as % of dry weight (n = 2). Each sample consists of 3 pooled roots. Vertical bars correspond to the standard deviation of the average.

Sugar content and composition as well as starch content were analysed in tap-roots from the two species (Figure 3). Sugar beet and parsnip tap-roots were shown to have different storage compound composition.

Sugar beet was almost exclusively storing sucrose with only very small proportions of the dry weight as hexoses and potential starch whilst parsnip stored appreciable amounts of starch, sucrose and hexoses.

Levels of sugars (glucose, fructose and sucrose) and starch were measured in sugar beet root and parsnip tap-root and calculated as percentage of dry weight matter. No difference was found between samples of light and dark sampled tap-roots from the same developmental time point. Therefore the results were combined to sugar beet 41 DAP, sugar beet 54 DAP, parsnip 48 DAP and parsnip 61 DAP. The measured starch of sugar beet was found to be insignificant at around 1% of the dry weight at both time points. Sugar content increased from 48% at 41 DAP to 56% of dry weight matter at 54 DAP. More than 98% of the sugar in sugar beet tap-root was sucrose and only very small amounts of fructose and glucose could be detected (Figure 3). The parsnip tap-root starch content increased from 21% at 48 DAP to 33% of dry weight matter at 61 DAP. The sugar content of parsnip tap-roots was relatively constant, around 15% of dry weight, for both samplings (Figure 3).

In addition to sucrose, hexoses were found at significant levels in parsnip. Among sugars the proportion of sucrose increased from 56% to 72% at the second time point. As a result hexose proportions decreased with development, glucose from 19% to 12% and fructose from 25% to 16% of total sugars.

### Protein extraction

Soluble proteins were extracted from sugar beet and parsnip tap-roots for further analysis of enzyme activities involved in starch biosynthesis. Protein concentrations of fresh weight were approximately twice as high for parsnip samples compared to sugar beet samples with small fluctuations between harvest time points (results not shown).

### Starch biosynthetic enzyme activities in sugar beet and parsnip tap-roots

Activities of the main enzymes in the starch biosynthetic pathway were investigated in tap-root crude protein extracts from parsnip and sugar beet. Four enzymes are critical in the building of branched α-glucans in amyloplasts; phosphoglucomutase (PGM), ADP-glucose pyrophosphorylase (AGPase), starch synthase (SS) and starch branching enzyme (SBE). Activities could be detected for all four enzymes in both parsnip and sugar beet tap-root samples (Table 1). Due to differences in the level of extractable protein from the different species, observed fluctuations in enzyme activity levels were more obvious per fresh weight level than per protein level.

**Table 1:** Starch biosynthetic enzyme activity in soluble protein extracts from parsnip and sugar beet tap-roots

	**PGM**	**AGPase**	**SS**	**SBE**
**Root tissue and developmental stage**	**(Units converting 1 μmole G1P to G6P, μg soluble protein**^**-1**^**, min**^**-1**^**)**	**(nmol ADP-glucose, μg soluble protein**^**-1**^**, min**^**-1**^**)**	**(nmol ADP-glucose converted to starch, μg soluble protein**^**-1**^**, min**^**-1**^**)**	**(nmol G1P converted to branched starch, μg soluble protein**^**-1**^**, min**^**-1**^**)**
Parsnip 48 DAP (light)	0.09 ± 0.02a	0.006 ± 0.002a	0.46 ± 0.20a	0.33 ± 0.01ab
Parsnip 48 DAP (dark)	0.12 ± 0.06a	0.007 ± 0.003a	0.55 ± 0.18a	0.38 ± 0.03ab
Parsnip 61 DAP (light)	0.12 ± 0.06a	0.006 ± 0.002a	0.68 ± 0.30a	0.52 ± 0.10a
Parsnip 61 DAP (dark)	0.11 ± 0.06a	0.007 ± 0.003a	0.71 ± 0.10a	0.47 ± 0.02a
Sugar beet 41 DAP (light)	0.09 ± 0.02a	0.007 ± 0.001a	0.43 ± 0.13a	0.20 ± 0.07b
Sugar beet 41 DAP (dark)	0.11 ± 0.03a	0.008 ± 0.001a	0.41 ± 0.06a	0.22 ± 0.04b
Sugar beet 54 DAP (light)	0.09 ± 0.02a	0.006 ± 0.001a	0.38 ± 0.06a	0.29 ± 0.17ab
Sugar beet 54 DAP (dark)	0.07 ± 0.004a	0.007 ± 0.001a	0.50 ± 0.16a	0.22 ± 0.08b

Samples were taken from parsnip tap-roots harvested 48 and 61 days after planting (DAP) and sugar beet tap-roots harvested 41 and 54 DAP. Enzyme activity of phosphoglucomutase (PGM), ADP-glucose pyrophosphorylase (AGPase), starch synthase (SS) and starch branching enzyme (SBE) were measured on crude extracts. Data are means ± SD of values from extracts derived from different homogenates of 3 pooled roots (n = 3). Columns sharing the same letters were not significantly different according to Tukey’s test (P = 0.05).

Phosphoglucomutase activity can be found in the cytosol and the plastid. PGM activity was detected in both sugar beet and parsnip tap-root at similar activities per protein level (Table 1). It could not be determined whether the activity was originating from the cytosol and/or the plastid.

The AGPase enzyme activity was similar between the parsnip and sugar beet samples with regards to enzyme activity per protein level (Table 1). No alteration in AGPase activity could be found between the different time points of harvest.

Parsnip and sugar beet upheld comparable levels of starch synthase per protein level leading to precipitable α- glucans (Table 1).

All samples of respective species displayed starch branching enzyme activity. SBE activity levels per μg protein ranged for sugar beet from 38% to 61% of the levels found in parsnip. The late parsnip harvests displayed in general a higher starch branching enzyme activity level than sugar beet (Table 1).

### Expression of genes important for starch accumulation

**Figure 4:**
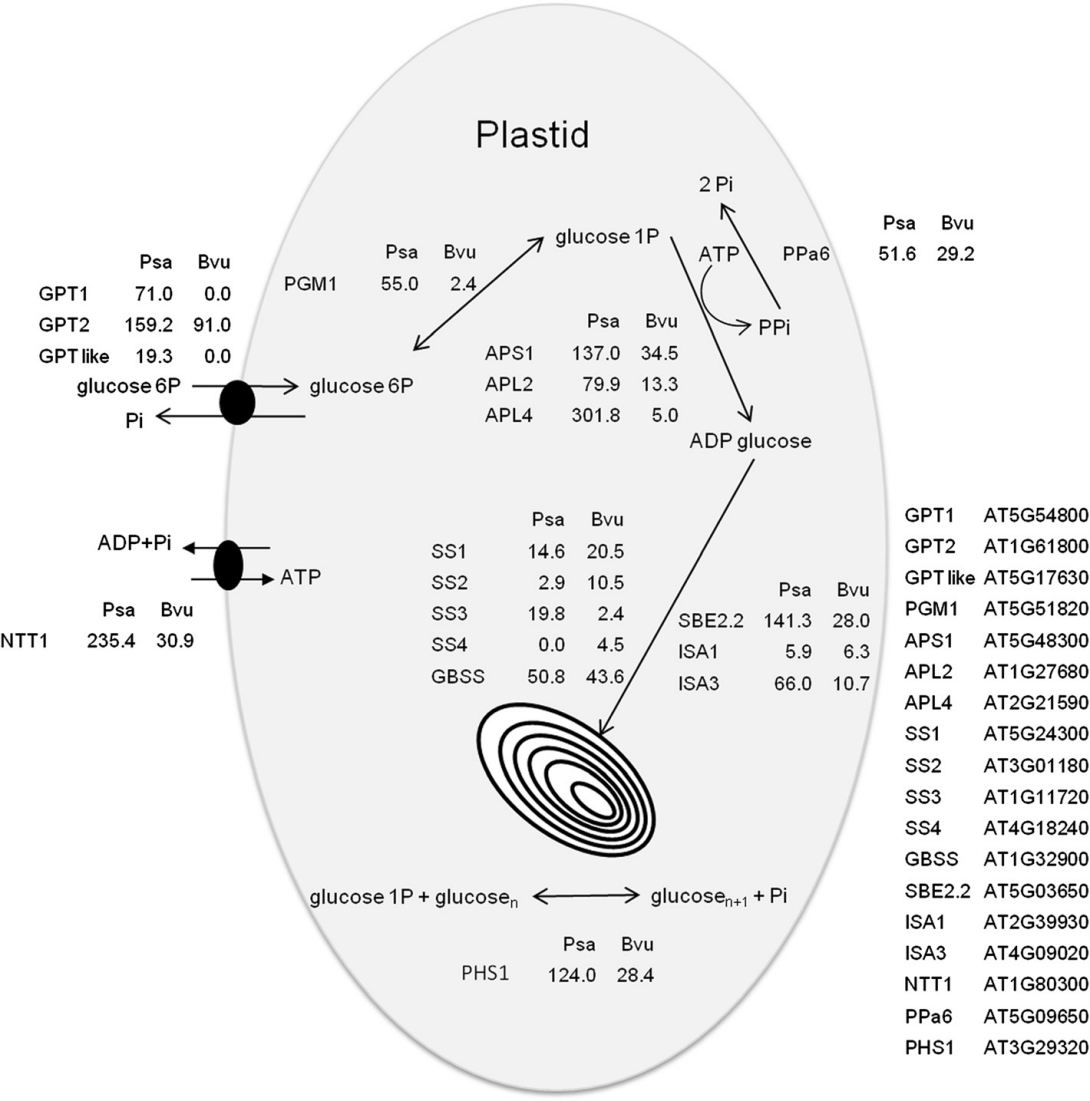
**Expression levels of parsnip and sugar beet genes encoding functions in starch accumulation.** Number of tap-root Illumina HiSeq 2000 reads per million reads (RPM) mapped on reference assemblies of *P. sativa* (Psa) and *B. vulgaris* (Bvu) corresponding to different cDNAs of functions involved in starch accumulation. The closest homologous *Arabidopsis thaliana* loci by BLASTx are given in the figure. GPT – glucose phosphate transporter, PGM1 – plastidic phosphoglucomutase, APS – ADP-glucose pyrophosphorylase small subunit, APL – ADP-glucose pyrophosphorylase large subunit, SS – soluble starch synthase, GBSS – granule bound starch synthase, SBE – starch branching enzyme, ISA – isoamylase, PHS – starch phosphorylase, NTT – ATP/ADP translocator and PPa6 – plastidic pyrophosphorylase.

Transcriptomes of root tissue in an active storage accumulation phase, sugar beet (54 DAP) and parsnip (61 DAP), were compared between sugar beet and parsnip. This analysis showed that all major genes coding for starch biosynthetic enzymes or genes coding for hexose-phosphate conversion were expressed in sugar beet tap-root even though no starch was produced (Figure 4).

Phosphoglucomutase (PGM) exists in both cytosolic and plastidic forms which are derived from different genes, where the plastidic form has been shown to be of importance for starch synthesis of dicots [21,22]. The analysis of transcriptome data indicated that the plastidic form of PGM was 23 fold more abundant in parsnip as compared to sugar beet (Figure 4). Genes coding for the large isoform of ADP-glucose pyrophosphorylase (AGPase, APL) were found to be much less expressed in sugar beet than in parsnip. Furthermore, more transcripts for the small subunit (APS) than for the large subunit were found in sugar beet, which is contrary to the situation in parsnip where more mapped reads were found for the large subunit (Figure 4). The ratio between large and small subunit transcripts was found to be 2.79 in parsnip and 0.53 in sugar beet. In sugar beet, mainly starch synthase 1 and 2 (SS1 and SS2) were found to be expressed whereas in parsnip, it was mainly starch synthase 1 and 3 (SS1 and SS3). Granule bound starch synthase (GBSS) was found expressed at a similar level in sugar beet as compared to parsnip. For sugar beet as well as parsnip, expression of genes for starch branching enzyme 2 (SBE2.2) but not of branching enzyme 1 (SBE1) could be found in the tap-roots. Genes important for starch tuning, isoamylases (ISA1 and ISA3) as well as starch phosphorylase (PHS1), were found expressed in both species. ISA1 was expressed at comparable levels but ISA3 (6-fold) and PHS1 (4-fold) was more abundant in parsnip. Genes encoding support activities for starch synthesis were generally found to be more highly expressed in parsnip. In total three genes encoding putative glucose phosphate transporters (GPT) could be identified in parsnip tap-root of which two forms completely lacked expression in sugar beet tap-root. One gene encoding an ATP/ADP translocator (NTT1) was found to be expressed in both species but 8 fold more abundant in parsnip (Figure 4). A gene encoding a plastidic pyrophosphorylase (PPa6) was found to be slightly more abundant in parsnip, with a ratio close to 2:1. Expression levels of genes typically associated with starch degradation and hydrolysis were also investigated (Table 2). More isoforms of genes encoding α- amylase and β-amylase were expressed in parsnip and for β-amylase there was a difference in which isoforms were more highly expressed for sugar beet and parsnip respectively. Summed up, a higher expression was found in parsnip for genes encoding α- and β-amylases. Genes encoding α-glucan, water dikinase/phosphoglucan, water dikinase (GWD/PWD) and disproportionating enzyme (DPE) were more highly expressed in parsnip.

**Table 2:** Expression levels of genes encoding functions in starch degradation in parsnip and sugar beet tap-root

	**Psa**	**Bvu**	
GWD/PWD	45.8	23.3	AT1G10760
GWD/PWD	19.4	2.6	AT5G2670
α-amylase	23.9	0.0	AT4G25000
α-amylase	10.0	7.1	AT1G76130
α-amylase	19.5	0.0	AT1G69830
β-amylase	62.7	1.2	AT3G23920
β-amylase	14.3	11.4	AT4G00490
β-amylase	3.2	17.7	AT5G18670
β-amylase	22.5	0.0	AT2G45880
β-amylase	4.7	22.9	AT4G17090
DPE1	42.6	13.1	AT5G64860
DPE2	17.1	14.4	AT2G40840

Number of tap-root Illumina HiSeq 2000 reads per million reads (RPM) mapped on reference assemblies of *P. sativa* (Psa) and *B. vulgaris* (Bvu) corresponding to different cDNAs of functions involved in starch degradation. The closest homologous *Arabidopsis thaliana* loci by BLASTx are given in the table. GWD/PWD – α-glucan, water dikinase/phosphoglucan, water dikinase, DPE – disproportionating enzyme.

## Discussion

The aim of this investigation was to study carbon storage accumulation in developing sugar beet and parsnip tap-roots. Furthermore, the concomitant presence or lack of activity from starch biosynthetic enzymes or expression of their corresponding genes was studied, which potentially could explain the differential storage strategies among the two species. As parsnip partially stores starch while sugar beet stores sucrose, a comparison between these two species will provide a better understanding of carbon allocation in these underground storage tissues and also an understanding of the genetic and enzymatic factors governing the accumulation of the two different carbon storage compounds.

The two time points for sampling were chosen when the roots were assumed to be in an early accumulating phase, reassuring that the filling of the sink cells was ongoing. Sugar beet is stated to be fully active in a quite early stage [23]. Parsnip has to our knowledge not been investigated in this aspect. Generally parsnip germinated slower than the sugar beet plants which motivated the 7 days delayed harvest compared to sugar beet. Measurements confirmed that the plants were harvested in an ongoing accumulative phase.

In the early phase of root and tuber storage organ development, cells consist mainly of a large vacuole. The vacuole contains sugars which are used as energy and building blocks for cell proliferation and expansion. The small starch granules that are present in the juvenile root cells are at this stage displaced towards the cell walls. During development, cells change from structural expansion of the organ to storage accumulation and most cells switch to filling up storage reserves such as starch granules or oil droplets and the vacuole is gradually compressed [24-26]. Sugar beet cells in the tap-root seemingly differ from other typical underground storage organs and appear to stay in the juvenile storage organ phase with the vacuole filled with sucrose as main component of the cell.

Investigation of tissue samples provided visual evidence for presence or absence of starch granules in the tap-root cells. The observation of enlarged starch granules and reduced size of the vacuoles in parsnip tap-root cells during growth corroborates with active starch biosynthetic enzyme activities. Sugar beet, however, maintained their relative vacuole size whereas cell walls thickened with no visible starch granules formed.

Sucrose produced in photosynthetic source cells is transported to the sink cells where sucrose cleaving enzymes (sucrose synthase and invertase) convert the sucrose to hexoses in different subcellular compartments. The hexoses in the sugar beet tap-root are thought to be resynthesized to sucrose by sucrose phosphate synthase and sucrose phosphate phosphatase to be transported into the vacuole [9,27-29]. Our measurements of sugar composition showed almost exclusively sucrose accumulation with very low levels of hexoses in the developing sugar beet tap-roots. The very low levels of hexoses present or accumulated in sugar beet tap-root indicate that either the sucrose is processed very fast into storage sucrose or that there is a direct transport route for sucrose into the storage vacuole without prior degradation and re-synthesis of the transported sucrose.

In a typical starch accumulating plant, such as potato, the hexoses are transported as hexose phosphates into the amyloplast where it is utilized for the synthesis of starch. Starch accumulation is a response to sucrose availability and thus, no hexoses are stored in the potato tuber [30]. In our experiments, sugar composition in parsnip was distributed in more equal parts of hexoses and sucrose, suggesting that the parsnip tap-root is not a pure starch storing organ but something in between the sugar beet and a typical starch accumulating organ. The presence of hexoses in parsnip tap-root could reflect a less efficient starch synthesis and sucrose accumulation as compared to a typical sucrose or starch accumulating organ such as sugar beet and potato. The hexoses in parsnip tap-root might instead suggest an on-going interconversion between starch and sucrose.

Several genes involved in starch biosynthesis have been isolated and studied in sugar beet although this has been performed with regards to photosynthetic structures. Additional expressed genes in sugar beet that are involved in starch biosynthesis can be found from searching Expressed Sequence Tag (EST) databases. Structural studies of sugar beet leaf tissue support the presence of starch biosynthetic enzyme activities by illustrating the common diurnal ability of photosynthetic tissue to produce starch. However, according to our knowledge, until now there has been no study directed to the presence or lack of expressed genes or activity of enzymes involved in starch biosynthesis in sugar beet tap-root that could explain the complete absence of starch in these structures. The aim of our enzymatic studies was primarily to determine presence or absence of starch biosynthetic enzyme activity and, surprisingly, our studies showed that sugar beet root had good activities of major starch biosynthetic enzymes, but no starch was accumulated. The four enzymes PGM, AGPase, SS and SBE, which are taking part in synthesis of branched glucans, were all active in sugar beet tap-root and within the same order of magnitude as in parsnip tap-root. When comparing expression levels of genes encoding these key enzymes in starch synthesis in sugar beet and parsnip tap-root, it is evident that most of these genes were also expressed at levels within the same order of magnitude. Exceptions to this were the genes coding for plastidic phosphoglucomutase and the large subunit of ADP-glucose pyrophosphorylase. However, this does not explain the complete lack of visible starch granules in sugar beet tap-root. For example, mutants or silencing of these two genes as well as transgenic silencing studies of Arabidopsis and pea display low starch content, but not a complete loss of starch accumulation [31-33]. Similarly as found in this study, expression of some of the genes encoding for these key enzymes can be found as expressed in sugar beet when assessing supplementary information of Bellin et al. [20]. Generally, support functions for starch synthesis such as genes encoding transporters for hexose phosphates and energy in the form of ATP were found to be less expressed in sugar beet than in parsnip tap-root although a complete lack of expression only was seen for a couple of transcripts. One example is genes coding for proteins with putative glucose 6-phosphate transport function where it is not yet fully deciphered which genes exactly are encoding the various transport functions. Sugar beet tap-root was found to completely lack mapped reads to transcripts corresponding to two forms which could be predicted to have related transport functions but where expression could be found in parsnip tap-root.

A number of studies of genes and enzymes of importance for synthesising the branched polymeric structure of α-1,4 linkages with α-1,6 branches have been published. Starch granule formation is more complex than production of long branched glucose polymers. To organize the long and branched glucose molecules into well-organized granules, debranching activities are needed for trimming the glucans and thus structuring the granule [34-36]. The role of debranching activities is not fully understood but it has been shown that in sugary-1 mutant maize endosperm a deficiency of debranching enzymes is proportional to phytoglycogen accumulation, a highly branched water soluble glucan [37]. Expression of genes encoding isoamylases which have been found to be key enzymes for proper starch granule formation were found in this study to be expressed in sugar beet as well as parsnip tap-root. Debranching and other starch hydrolyzing enzyme activities have previously been reported and characterized in sugar beet tap-root [38,39]. From this information it could also be speculated that the lack of starch accumulation in sugar beet tap-root could be due to high expression of genes for α-glucan or starch degrading enzymes. Examination of transcripts for genes encoding enzymes associated with starch degradation and hydrolysis revealed lower levels in sugar beet compared to parsnip which in case of the opposite could have been an indication of a rapid turnover of any starch formed.

Even in root crops considered as non-starchy, such as carrot, starch is accumulated [40]. Indeed it is intriguing that such an extent of expression and activity related to starch pathways are present in sugar beet tap-root without starch produced.

## Conclusion

In conclusion, gene expression and enzymatic activities could be found for the major participants in starch biosynthesis in sugar beet, despite that structural analyses and chemical analysis failed to indicate any presence of starch. Even though some genes were found to be less expressed in sugar beet tap-root, a complete lack of starch granules cannot be explained by these results. Thus, there must be another mechanism or mechanisms which prevent sugar beet from producing starch in the tap-root, a default storage compound for underground sink organs. Starch is an energy-efficient storage form due to the insoluble starch granule compared to the soluble sucrose. During the storage phase from one year to the next, sugar beet tap-root needs to maintain an energy potential in order to keep the high concentration of sucrose in the vacuole, 500 mM sucrose in comparison to the 75 mM sucrose in the cytosol [12]. This apparent energy-demanding storage strategy based on sucrose could have evolved as a consequence of the saline growing conditions of the ancestor sea beet, where high sucrose concentration could be of importance for keeping salt out of the cells. Thus, sugar storage in sea beet may have evolved as a result of its environmental adaptation from a starch accumulating tap-root ancestor. The general expression of genes and activity of enzymes in the starch biosynthetic pathway in the sugar beet tap-root could thus be regarded as a genetic relict with no present functions.

## Methods

### Plant material

Sugar beet seeds (*Beta vulgaris ssp. altissima,* “Balder”) and sea beet (*Beta vulgaris ssp. maritima*) were kindly provided by Nordic Genetic Resource center, Alnarp, Sweden.

Parsnip seeds (*Pastinaca sativa* “White Gem”) were purchased online from Impecta Fröhandel, Julita, Sweden, http://www.impecta.se.

### Growth conditions, fresh and dry weight

The parsnip and sugar beet seeds were sown in 2 litres pots in greenhouse during spring. The plants were regularly fertilized and watered. Leaf and tap-root samples were taken at 2 time points, at each time point roots sampling was performed both at the end of the light period and at the end of the dark period. Parsnip was sampled at 48 and 61 days after planting (DAP). Sugar beet was sampled at 41 DAP and 54 DAP. The primary root fresh weight was measured. Dry weight determination was performed by freeze drying the roots (n > =3) until no weight change was noted (≈72 hrs). Plant tissues were frozen in liquid nitrogen and stored at −80°C for further studies.

Sea beet was cultivated in an aeroponic system [25] and samples were taken after 3 months.

### Structural studies

#### Fixation and plastic embedding

Fixation and plastic embedding of fresh roots and leaves of parsnip and sugar beet was performed as previously described [25].

#### Overview staining of sections

Triple staining methylene blue-azur A-safranin O (MAS), visualising proteins, lipids and starch, was performed to obtain an overview of the embedded tap-root tissue [41,42].

#### Starch staining of sections

In order to stain starch, the leaf sections were covered with 50% Lugol’s solution (Scharlau, Barcelona, Spain), for 1 min, rinsed with water, air-dried, and mounted with Biomount (British Biocell, Cardiff, UK).

MAS and Lugol’s stained sections were studied in a light microscope (Leica Microsystems, Wetzlar, Germany).

#### Starch staining of homogenized tissue

Homogenized sea-beet tap-root tissue was spread on a microscope slide and Lugol’s solution was added. The stained tissue was studied in a light microscope (Leica Microsystems, Wetzlar, Germany).

#### β-glucans

The fluorochrome Calcofluor White (Fluorescent brightener 28, Sigma Aldrich, St. Louis, MO, USA) was used to visualise β-glucans at 420 nm [43,44]. Sections were covered with 0.0001% Calcofluor for 10 min, rinsed with dH_2_O and mounted with Mowiol 4–88 (La Jolla, CA, USA) [45] to be studied in a fluorescence microscope (Leica Microsystems, Wetzlar, Germany). As a control autofluorescence of unstained sections was studied.

### Starch and sugar analysis

Since the measurements were made on whole root homogenates a minor part of the obtained values derives from non-storage parts of the cell such as cell-walls or non-storage cellular compartments. Also, when measuring starch there is a possibility that other α-glucans, for instance phytoglycogen, are included in the assay by the method used.

The analysis is a standardized method, SCAN-P 91:09, recommended to be used by Scandinavian pulp, paper and board industry.

Sugar beet and parsnip root tissue were freeze dried and homogenized by grinding in a mortar. For the starch assay the homogenate was dissolved in an appropriate volume ddH_2_O and hydrolysed to glucose in a two-step enzymatic process [46]. The glucose was subsequently detected on an ion exchange chromatograph (Bioscan, Metrohm, Herisau, Switzerland) Colonn Metrosep Carb1, injectionvolume 6 μl, eluent 0.2 M NaOH, flowrate 1 ml/min, ambient temperature, detector PAD (pulsed amperometric detection). The assay measures the total amount of α-glucans e.g. starch and phytoglycogen in a sample.

For the sugar analysis, 100 mg freeze dried homogenate was dissolved in 1 ml 80% EtOH and extracted at −20°C for two weeks. Analysis of sugars was made on an ion exchange chromatograph (Bioscan, Metrohm, Herisau, Switzerland) using the same setup and procedure as the starch analysis. The analysis was performed with sugar solutions of known concentration and composition as standards.

### Protein extraction and determination

Crude protein extracts were obtained by homogenizing root tissue in a mixer mill (MM400, Retsch GmbH, Haan, Germany) in a stainless steel container, pre-chilled in liquid nitrogen to keep the tissue frozen and the enzyme activity intact. Protein was extracted from the fine powder according to a modified protocol which excludes BSA from the extraction buffer [47]. The extracts were divided in aliquots, snap-frozen in liquid nitrogen and stored at −80°C. Protein concentrations were determined by BCA Protein Assay – Reducing agent compatible (Pierce, Rockford, IL, USA).

### Assays for starch biosynthetic enzymes

#### Phosphoglucomutase

PGM activity was determined in a spectrophotometric coupled assay. Conversion of glucose-1-phosphate (G1P) is catalyzed by PGM and the resulting glucose-6-phosphate (G6P) is subsequently catalyzed by glucose-6-phosphate dehydrogenase to 6-phosphogluconate. In parallel with the second reaction, NADP is reduced to NADPH and the reaction is measured at 340 nm [48]. Extract corresponding to 20 μg crude protein was added to a substrate solution and the change in absorbance at 340 nm was measured after 2, 5, 10, 15 and 25 minutes. A standard curve was made by assaying various concentrations of phosphoglucomutase (Phosphoglucomutase from rabbit muscle, P3397, SIGMA Aldrich, St. Louis, MO, USA) under the same conditions as the samples. The specificity of the assay was tested by excluding G1P from the substrate. Enzyme activity was calculated as G1P converted to G6P (μmol) by soluble crude protein (ng) per minute.

#### ADP-glucose pyrophosphorylase

AGPase activity was determined [49] on 20 μg crude protein. The samples were measured after 0, 30 and 90 minutes.

AGPase catalyzes the reaction conversion of ATP and G1P to ADP-glucose and pyrophosphate (PPi). The assay measures phosphate after splitting produced PPi by inorganic pyrophosphatase. A standard curve for phosphate was made by mixing various concentrations of KH_2_PO_4_ with Mg-Am stain and following the measuring procedure as in the assay. Phosphate content in crude protein extract was measured by inactivating the crude enzyme extract at 60°C for 10 min and then measuring the samples as described for the standard curve. The background content of pyrophosphate was measured by incubating the inactivated crude extract with inorganic pyrophosphatase and then assaying phosphate content same procedure as the standard curve. Enzyme activity was calculated as produced ADP-glucose (nmol) per soluble crude protein (μg) per minute.

The specificity of the assay was examined by excluding G1P and ATP from the substrate both separately and in combination to determine and exclude the cytosolic UDP-glucose pyrophosphorylase activity.

#### Soluble starch synthase

Crude root protein extract (10 μg) was assayed for starch synthase activity. Activity was calculated by measurements after 0, 30, 90 and 120 minutes. The starch synthase assay was performed as previously described but with a small modification, where amylopectin in the substrate solution was exchanged to glycogen [50]. The reaction was terminated at 95°C for 2 minutes, and precipitated and washed according to the protocol and dissolved in 1 ml ddH_2_O. Five ml scintillation mix (Ultima-Flo M, Packard, Perkin Elmer, Waltham, MA, USA) was added to 0.5 ml of the dissolved starch product and radioactivity was measured in a liquid scintillation counter (Philips PW 4700, Eindhoven, The Netherlands). The starch synthase activity was calculated as the amount ADP-glucose converted to starch per minute and μg total protein.

#### Starch branching enzyme

Crude protein extract (10 μg) was assayed for starch branching enzyme activity [51] with some modifications. Glycogen (3 μg) was added to the substrate as a primer to the glucan chain. Control reactions were performed excluding phosphorylase a to leave out possible endogenous phosphorylase activity in the extracts. Precipitation, dissolving and counting of radioactivity was performed as described in the starch synthase assay. Activity was calculated by measurements after 0, 60 and 90 minutes. The starch branching enzyme activity was calculated as the amount glucose-1-phosphate converted to branched starch per minute and μg total protein.

### Transcriptome sequencing and analysis

#### Total RNA extraction

Samples were chosen from the second sampling of sugar beet (54 DAP) and parsnip (61 DAP). Total RNA was extracted from a homogenate of three pooled individuals of root or leaf tissue respectively with Plant RNA Reagent (Invitrogen, Life technologies Ltd, Carlsbad, CA, USA). Concentration was measured on a NanoDrop (NanoDrop™ 1000 Spectrophotometer, Thermo Scientific, Waltham, MA, USA) and quality was confirmed on a 1.2%, E-gel (Invitrogen, Life Technologies Ltd, Carlsbad, CA, USA).

#### cDNA library synthesis

DNA sequencing and data processing was provided by Eurofins as a service. Two normalised random primed cDNA libraries were produced from pooled leaf and tap-root mRNA from sugar beet and parsnip respectively. These were subsequently subjected for sequencing using Roche GS FLX Titanium series chemistry at a scale of ½ segment of a full run for each cDNA library.

#### Trancriptome analysis

After quality analysis, passed reads were assembled into contigs and contigs collected in one reference file each for sugar beet and parsnip respectively. 531,058 passed reads were used for sugar beet and 563,841 reads were used for parsnip.

Two 3'-fragment cDNA libraries with bar-coded adaptors were produced from tap-root mRNA from sugar beet and parsnip respectively. These were subsequently subjected to sequencing using Illumina HiSeq 2000 technology utilizing one channel in total for both samples. 24,586,598 reads passed quality analysis for sugar beet and 39,749,856 reads for parsnip. As a next step Illumina reads were assembled and used to improve the reference files produced after the Roche GS FLX Titanium sequencing and assembly resulted in new reference files for both parsnip and sugar beet where contigs consisted of data from both sequencing sets.

Passed Illumina reads were mapped to the final reference files produced for sugar beet and parsnip. The number of reads mapped to each contig yielded an estimate of gene expression corresponding to the particular contig in comparison to number of reads mapped to other contigs. As only 3'-fragments were used for mapping, this by itself resulted in a normalization of the reads for each transcript. Gene expression for each transcript is thus expressed as the number of mapped reads to a specific contig per million reads (RPM).

## Abbreviations

ESTs: Expressed sequence tags; DAP: Days after planting; PGM: Phosphoglucomutase; AGPase: ADP-glucose pyrophosphorylase; SS: Starch synthase; SBE: Starch branching enzyme.

## Competing interests

This study has partially been financed by Lyckeby Stärkelsen Research Foundation, which is the research foundation of a commercial entity. This relation has not affected our interpretation of data or presentation of information.

## Authors’ contributions

HT designed and conducted the majority of the experimental work. MA contributed in designing and coordinating the project, edited and revised the manuscript, SM participated in the structural work and edited the manuscript, IT performed the sugar and starch analysis, PH contributed in designing and coordinating the project and conducted the transcriptome analysis. HT and PH wrote the manuscript. All authors read and approved the final manuscript.

## Supplementary Material

Additional file 1 **Sections of leaves illustrating diurnal changes.** Sections of leaves stained with Lugol’s solution illustrating diurnal changes. a. Sugar beet leaf sampled 12 hours after sunrise. Dark spots, indicated by arrows, show accumulated starch. b. Sugar beet leaf sampled in dark, no starch is detected, c. Parsnip leaves sampled 12 hours after sunrise. Starch is detected. d. Parsnip leaf sampled in dark, No starch is detected but chloroplasts are shown clearly. Scale bar 50 μm. Click here for file

Additional file 2 **Illustration of cambium rings.** Cambium rings of green house grown sugar beet roots 41 days after planting (a) and 54 days after planting (b). Sections are stained with Lugol’s solution. Scale bars 5 mm. Click here for file
