# Supplementary figures and images for: Starch biosynthetic genes and enzymes are expressed and active in the absence of starch accumulation in sugar beet tap-root
Source: BMC Plant Biol. 2014 Apr 23;14:104. doi: 10.1186/1471-2229-14-104 (PMC4108048; doi:10.1186/1471-2229-14-104)

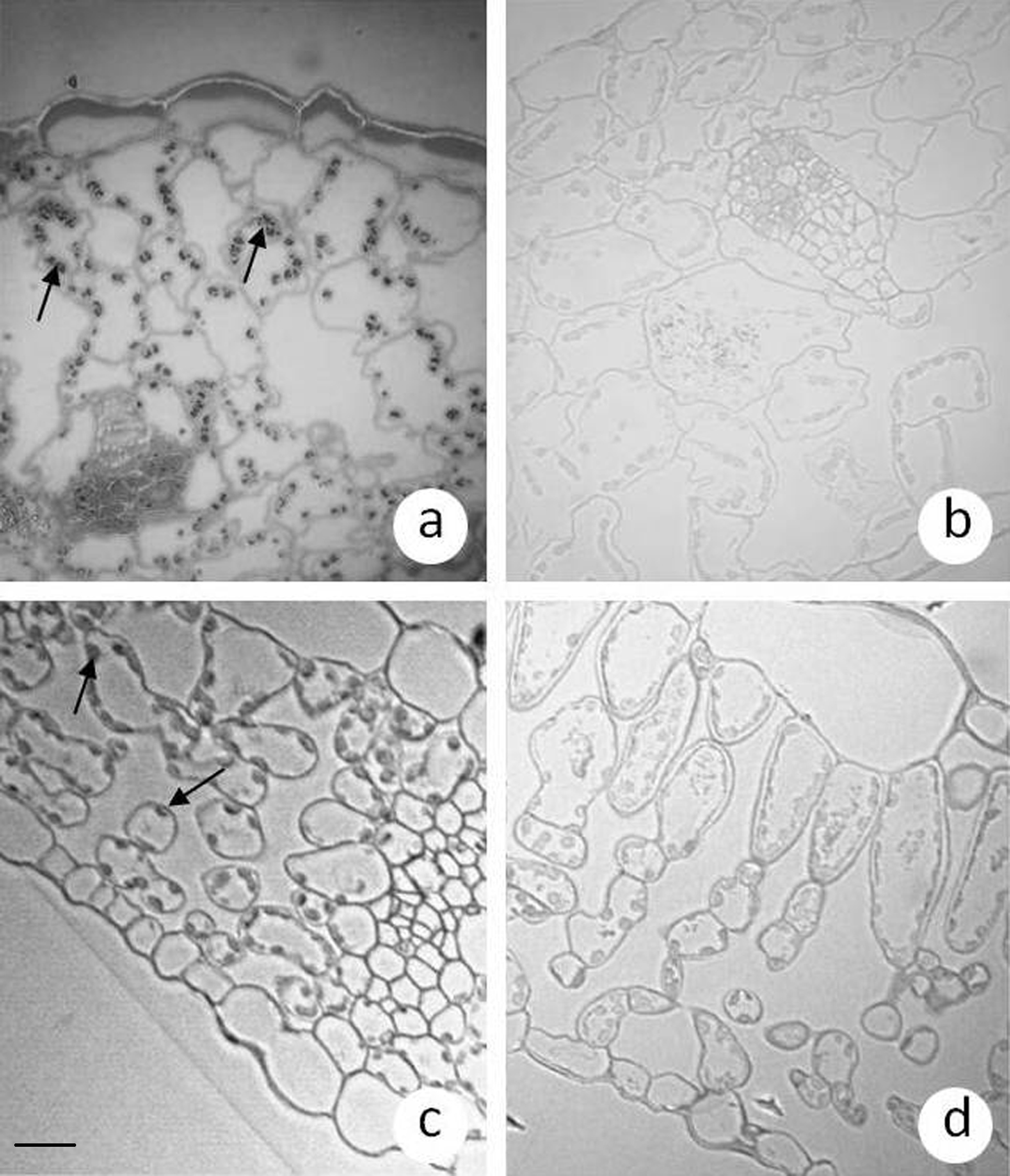

Supplement: Additional file 1 — Sections of leaves illustrating diurnal changes. Sections of leaves stained with Lugol’s solution illustrating diurnal changes. a. Sugar beet leaf sampled 12 hours after sunrise. Dark spots, indicated by arrows, show accumulated starch. b. Sugar beet leaf sampled in dark, no starch is detected, c. Parsnip leaves sampled 12 hours after sunrise. Starch is detected. d. Parsnip leaf sampled in dark, No starch is detected but chloroplasts are shown clearly. Scale bar 50 μm. [file 1471-2229-14-104-S1.tiff]

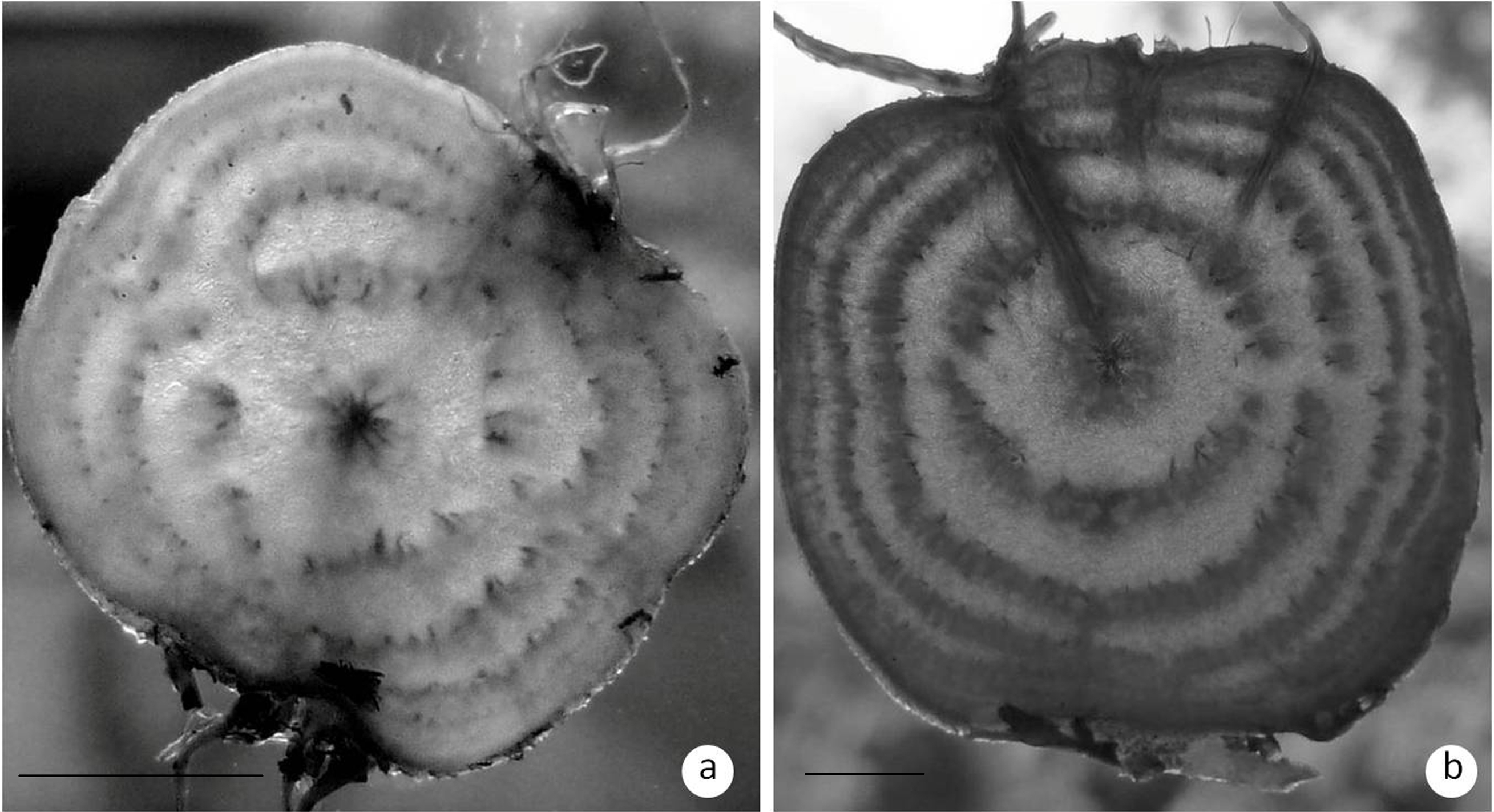

Supplement: Additional file 2 — Illustration of cambium rings. Cambium rings of green house grown sugar beet roots 41 days after planting (a) and 54 days after planting (b). Sections are stained with Lugol’s solution. Scale bars 5 mm. [file 1471-2229-14-104-S2.tiff]
